# Draft Genome Sequences of Several Fungal Strains Selected for Exposure to Microgravity at the International Space Station

**DOI:** 10.1128/genomeA.01602-16

**Published:** 2017-04-13

**Authors:** Nitin K. Singh, Adriana Blachowicz, Jillian Romsdahl, Clay Wang, Tamas Torok, Kasthuri Venkateswaran

**Affiliations:** a Jet Propulsion Laboratory, California Institute of Technology, Pasadena, California, USA; b Department of Pharmacology and Pharmaceutical Sciences, School of Pharmacy, University of Southern California, Los Angeles, California, USA; c Department of Chemistry, Dornsife College of Letters, Arts and Sciences, University of Southern California, Los Angeles, California, USA; d Lawrence Berkeley National Laboratory, Berkeley, California, USA

## Abstract

The whole-genome sequences of eight fungal strains that were selected for exposure to microgravity at the International Space Station are presented here. These baseline sequences will help to understand the observed production of novel bioactive compounds.

## GENOME ANNOUNCEMENT

In a screening project of natural products, fungal strains isolated from environments associated with the Chernobyl nuclear power plant (ChNPP) accident (1) have been investigated. The radiation-tolerant microorganisms selected for exposure to microgravity at the International Space Station were known to produce valuable natural products; their genomic sequences coded for secondary metabolism pathways; or they displayed positive radiotropism.

*Aspergillus niger*, an industrially important filamentous fungus, contains a sequence resembling the fumonisin gene cluster, which suggests that the fungus has the genetic potential to produce carcinogenic fumonisins (2). *A. niger* also produces an abundance of naphtho-gamma-pyrone secondary metabolites, which have been shown to have antibacterial, antifungal (3), antitumor (4), and cytotoxic (3, 4) activity.

*Aspergillus terreus* is used to produce organic acids, such as itaconic acid (5), or enzymes, such as xylanases (6, 7). One of the most important secondary metabolites made by *A. terreus* is the cholesterol-lowering molecule lovastatin. Discovery of this potent compound revolutionized the treatment of hypercholesterolemia (8, 9).

*Aureobasidium pullulans* is an important producer of pullulan, a homopolysaccharide of glucose that is widely used in the food, pharmaceutical, and electronics industries (10, 11). The whole-genome sequence of *A. pullulans* revealed significant biotechnological potential but also the presence of virulence factors that cannot be overlooked (12).

*Beauveria bassiana* is an entomopathogenic fungus used to produce biodegradable, nonpoisonous, and cost-efficient bioinsecticides (13). Genomic analysis of *B. bassiana* exhibits its capacity to produce a plethora of secondary metabolites, such as oosporein, bassianin, beauvericin, or oxalic acid (14). Beauvericin possesses antimicrobial, antiviral, antifungal, and antitumor activity (15).

*Cladosporium cladosporioides*, a ubiquitous organism, produces cladosporin and isocladosporin—secondary metabolites known to have antifungal activities. Its genome has not been sequenced yet (16, 17).

*Cladosporium sphaerospermum* is a plant endophyte but also an allergen to immunocompromised populations. It has the capacity to produce a variety of allergens, such as enolase, mannitol, dehydrogenase, and aldehyde dehydrogenase (18).

*Fusarium solani* is a plant pathogen that produces multiple phytotoxins, such as marticin, isomarticin, anhydrofusarubin, and javanicin, that cause sudden death syndrome of soybean (19), for example.

*Trichoderma virens* is a common rhizosphere fungus beneficial to plants and reported to induce a defense response of cotton to *Rhizoctonia solani*–incited seedling disease (20, 21).

The whole-genome sequences of these eight fungal strains were obtained by shotgun sequencing performed on an Illumina HiSeq2500 platform with a paired-end module. The NGS QC toolkit version 2.3 (22) was used to filter the data for high-quality vector- and adaptor-free reads for genome assembly (cutoff read length for high quality: 80%; cutoff quality score: 20). High-quality vector-filtered reads were used for assembly with the MaSuRCA genome assembler (*k*-mer size = 70) (23). Data from the final assembly of the strains, including number of scaffolds, total size, *N*_50_ contig length, G+C content, and GenBank accession numbers, are given in Table 1.

**TABLE 1:** Statistical summary for the eight draft fungal genome sequences

Strain	Strain designation	NCBI accession no.	Isolation location^a^	No. of contigs	Genome size (bp)	*N*_50_ (bp)	G+C content (%)
*Aspergillus niger*	JSC-093350089	MSJD00000000	ISS environmental surface	223	36,080,355	543,773	49.46
*Aspergillus terreus*	IMV 01167	MSJE00000000	Soil, Kirovograd region	331	31,580,414	482,632	52.24
*Aureobasidium pullulans*	IMV 00882	MSJF00000000	Wall surface, unit 4, ChNPP	879	40,984,331	98,085	51.01
*Beauveria bassiana*	IMV 00265	MSJG00000000	Wall surface, unit 4, ChNPP	735	35,190,057	138,299	51.72
*Cladosporium cladosporioides*	IMV 00236	MSJH00000000	Wall surface, unit 4, ChNPP	843	47,573,060	186,555	42.89
*Cladosporium sphaerospermum*	IMV 00045	MSJI00000000	Wall surface, unit 4, ChNPP	959	50,156,125	72,128	53.05
*Fusarium solani*	IMV 00293	MSJJ00000000	Wall surface, unit 4, ChNPP	876	51,318,644	812,484	47.58
*Trichoderma virens*	IMV 00454	MSJK00000000	Soil, 10-km ChEZ	197	42,025,033	1,319,489	48.44

a ISS, International Space Station; ChNPP, Chernobyl nuclear power plant; ChEZ, Chernobyl exclusion zone.

### Accession number(s).

This whole-genome shotgun project has been deposited in DDBJ/ENA/GenBank under the accession numbers given in Table 1. The versions described in this paper are the second versions.
